# Supplementary material for: Pyrazolo-triazolo-pyrimidine Scaffold as a Molecular Passepartout for the Pan-Recognition of Human Adenosine Receptors
Source: Biomolecules. 2023 Nov 3;13(11):1610. doi: 10.3390/biom13111610 (PMC10669182; doi:10.3390/biom13111610)
Supplement: Supplementary file 1 [file biomolecules-13-01610-s001.zip › SI_Biomolecules_R1_proof.pdf]

Supplementary Information

Pyrazolo-triazolo-pyrimidine Scaffold as a Molecular Passepartout for the Pan-Recognition of the Human Adenosine Receptors

Veronica Salmaso [1,*], Margherita Persico [2], Tatiana Da Ros [2], Giampiero Spalluto [2], Sonja Kachler [3], Karl-Norbert Klotz [3], Stefano Moro [1], Stephanie Federico[2,*]

Molecular Modeling Section (MMS), Dipartimento di Scienze del Farmaco, Università di Padova, via Marzolo 5, I-35131 Padova, Italy; veronica.salmaso@unipd.it; stefano.moro@unipd.it

[2] Dipartimento di Scienze Chimiche e Farmaceutiche, Università degli Studi di Trieste, Via Licio Giorgieri 1, I-34127, Trieste, Italy; sfederico@units.it; margherita.persico@phd.units.it; daros@units.it; spalluto@units.it

[3] Institut für Pharmakologie, Universität of Würzburg, Versbacher Str. 9, D-97078, Würzburg, Germany; sonja.kachler@uni-wuerzburg.de; klotz@toxi.uni-wuerzburg.de

* Correspondence: V.S. veronica.salmaso@unipd.it; Tel.: +39 049 827 5801; S.F. sfederico@units.it; Tel: +39 040 558 3671

Page 2	Table S1: Adenosine Receptors sequence identity and similarity.
	Figure S1: Alignment between hA$_{2B}$ and hA$_{2A}$ sequences for homology modelling.
	Figure S2: Alignment between hA$_3$ and hA$_1$ sequences for homology modelling.
Page 3	Figure S3: Binding mode of compound ZM-241385 at A$_{2A}$ AR in X-ray structure 4EIY.<br>Figure S4: Sequence Alignment of all Adenosine Receptors.
Page 4	Figure S5: Alternative docking pose of compound **11** at hA$_1$, hA$_{2A}$, hA$_{2B}$, hA$_3$ ARs.
	Labels Videos S1, S2, S3, S4

Table S1. Sequence identity and similarity among hA_1, hA_{2A}, hA_{2B} and hA_3 ARs. Full-sequence data are reported on the lower left (white background cells), and transmembrane helices (TM) data on the upper right (grey background cells).

	A1AR	A2A AR	A2B AR	A3 AR
A1AR	-	I 55%, S 70%	I 52%, S 70%	I 56%, S 77%
A2A AR	I 38%, S 48%	-	I 66%, S 82%	I 47%, S 66%
A2B AR	I 43%, S 59%	I 45%, S 56%	-	I 45%, S 64%
A3 AR	I 46%, S 65%	I 30%, S 45%	I 35%, 53%	-

Data were retrieved from the GPCRdb web site (https://gpcrdb.org).

Figure S1

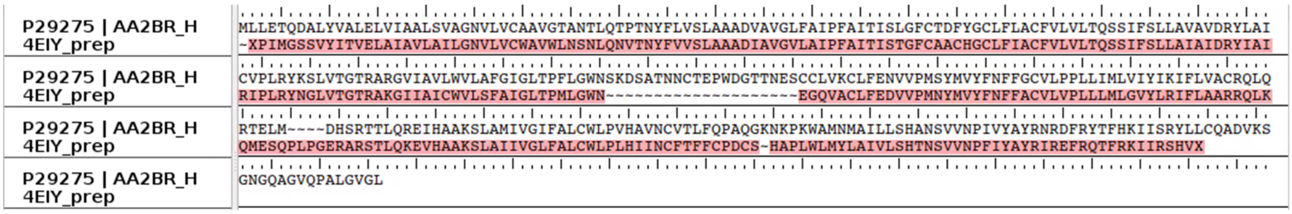

Figure S1. Sequence alignment between hA_{2B} AR and hA_{2A} AR, from structure 4EIY (after reconstruction of missing atoms and retro mutation to wild-type). Extracellular loop 2 was removed before the alignment. Alignment used for homology modeling.

Figure S2

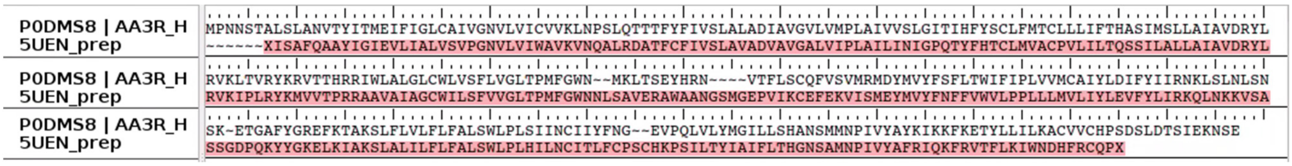

Figure S2. Sequence alignment between hA_3 AR and hA_1 AR, from structure 5UEN (after reconstruction of missing atoms and retro mutation to wild-type). Alignment used for homology modeling.

Figure S3

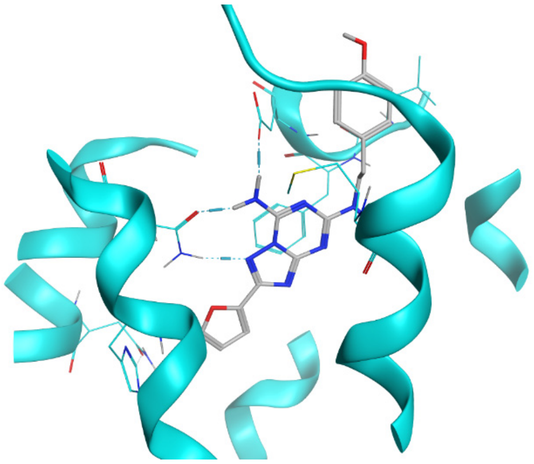

Figure S3. Binding mode of ZM-241385 (light grey) at hA_{2A} AR (cyan) in X-ray structure 4EIY.

Figure S4

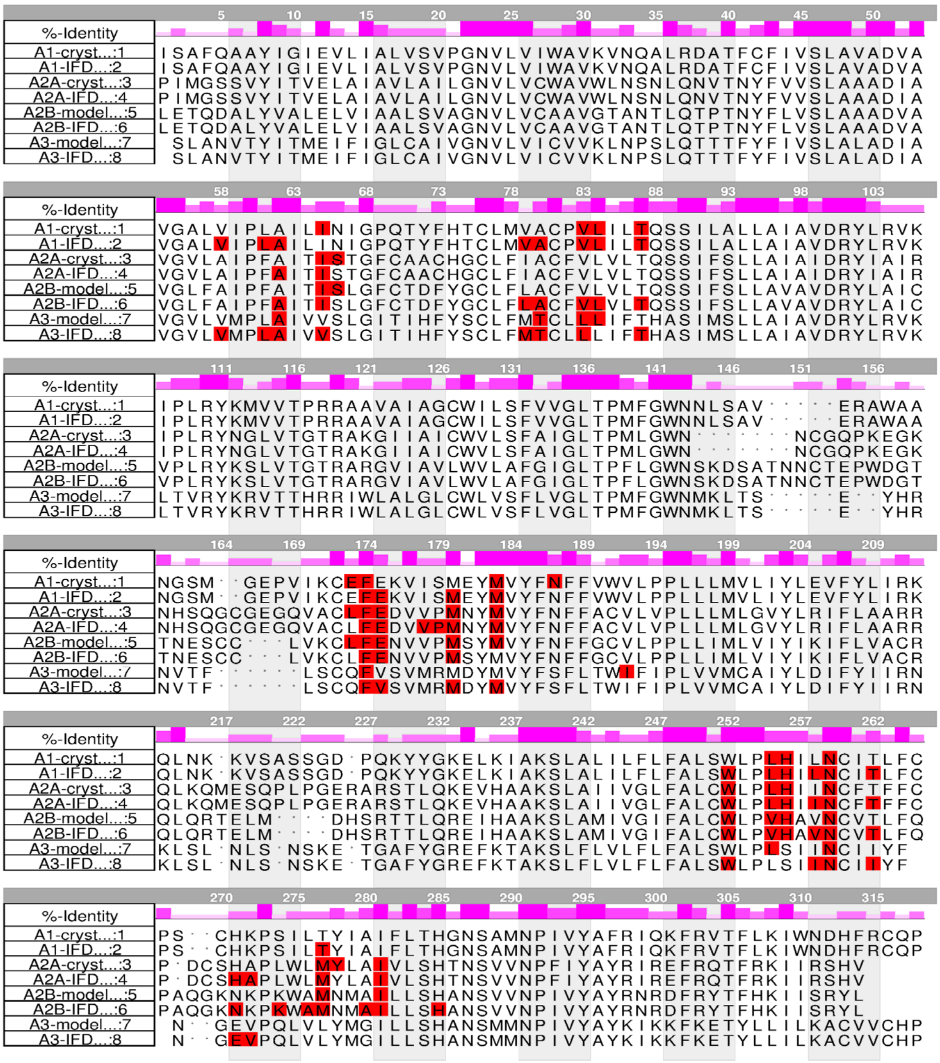

Figure S4. Sequence alignment from structure superposition of hA_1, hA_{2A}, hA_{2B} and hA_3 ARs. Magenta histograms represent % Identity. Residues of the binding site (within 3 Å from the predicted pose of compound **4** or **11**) are highlighted in red. To identify the binding site, 2 structures have been considered for each receptor: X-ray structure/homology model bound to compound **11** and Induced Fit refined structure bound to compound **4**.

Figure S5

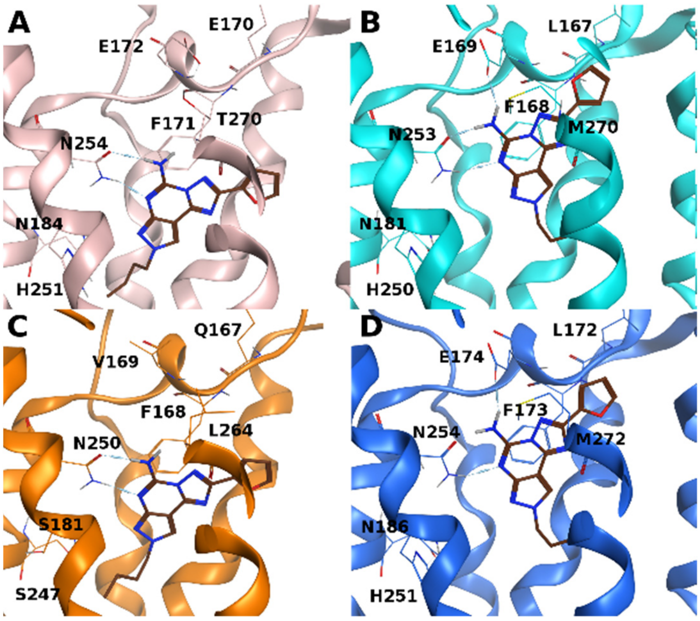

Figure S5. Docking pose of compound **11** (brown) at hA_1 (pink, **A**), hA_{2A} (cyan, **B**), hA_3 (orange, **C**), hA_{2B} (blue, **D**) ARs structures. The following receptor 3D structures were employed: experimental X-ray for hA_1 (PDB IDs: 5UEN) and hA_{2A} ARs (PDB IDs: 4EIY), homology models for hA_{2B} and hA_3 ARs (built on hA_{2A} and hA_1, respectively). A docking pose alternative to the ZM-241385-like one is displayed here.

Video S1.
Selected docking poses for compounds **1-12** (magenta) at hA_1 AR (pink) structure (PDB ID: 5UEN). Two alternative binding modes are shown for compounds **8**, **9**, **11** (z = ZM-241385-like; a = alternative). Compounds **8-12** were docked at the X-ray structure, compounds **1-7** were docked at the same structure refined by induced fit docking of compound **4**.

Video S2.
Selected docking poses for compounds **1-12** (magenta) at hA_{2A} AR (cyan) structure (PDB ID: 4EIY). Two alternative binding modes are shown for compounds **8**, **9**, **10, 11, 12** (z = ZM-241385-like; a = alternative). Compounds **8-12** were docked at the X-ray structure, compounds **1-7** were docked at the same structure refined by induced fit docking of compound **4**.

Video S3.
Selected docking poses for compounds **1-12** (magenta) at hA_{2B} AR (blue) structure (model built using hA_{2A} AR structure 4EIY as template). Two alternative binding modes are shown for compounds **8**, **9**, **10, 11, 12** (z = ZM-241385-like; a = alternative). Compounds **8-12** were docked at the homology model, compounds **1-7** were docked at the same structure refined by induced fit docking of compound **4**.

Video S4.
Selected docking poses for compounds **1-12** (magenta) at hA_3 AR (orange) structure (model built using hA_1 AR structure 5UEN as template). Two alternative binding modes are shown for compounds **8**, **9**, **11, 12** (z = ZM-241385-like; a = alternative). Compounds **8-12** were docked at the homology model, compounds **1-7** were docked at the same structure refined by induced fit docking of compound **4**.
